# P-447. Differences in Invasive Pneumococcal Disease Clinical Presentations and Serotype Distribution among Children with and without Underlying Risk Factors

**DOI:** 10.1093/ofid/ofaf695.662

**Published:** 2026-01-11

**Authors:** Kristina G Hulten, William J Barson, Philana L Lin, Steven Dahl, John S Bradley, Tina Q Tan, Pia Pannaraj, Jennifer Dien Bard, Kacy A Ramirez, Lindsay Grant, Adriano Arguedas, Maria J Tort, Ashley Miller, Alejandro D Cane, Bradford Gessner, Sheldon L Kaplan

**Affiliations:** Baylor College of Medicine, Houston, TX; Ohio State University College of Medicine and Public Health and Nationwide Children's Hospital, Columbus, Ohio; UPMC Children's Hospital of PIttsburgh, Pittsburgh, Pennsylvania; University of Arkansas for Medical Sciences, Little Rock, Arkansas; University of San Diego School of Medicine, Rady Children's Hospital, San Deigo, CA; Feinberg School of Medicine, Northwestern University, Chicago, Illinois; University of California San Diego; 8Children's Hospital Los Angeles; University of Southern California, Los Angeles, CA; Wake Forest School of Medicine, Oak Ridge, North Carolina; Pfizer Inc., Collegeville, PA; Pfizer, Collegeville, Pennsylvania; Pfizer, Inc, Collegeville, Pennsylvania; Pfizer, Collegeville, Pennsylvania; Pfizer, Collegeville, Pennsylvania; Pfizer, Inc., New York, New York; Baylor College of Medicine, Houston, TX

## Abstract

**Background:**

Following the introduction of routine administration of pneumococcal conjugate vaccines (PCV) to infants, more than half of children with invasive pneumococcal disease (IPD) in our surveillance study now have an underlying condition. We investigated IPD among children with and without underlying conditions to further characterize these groups.

**Table.**
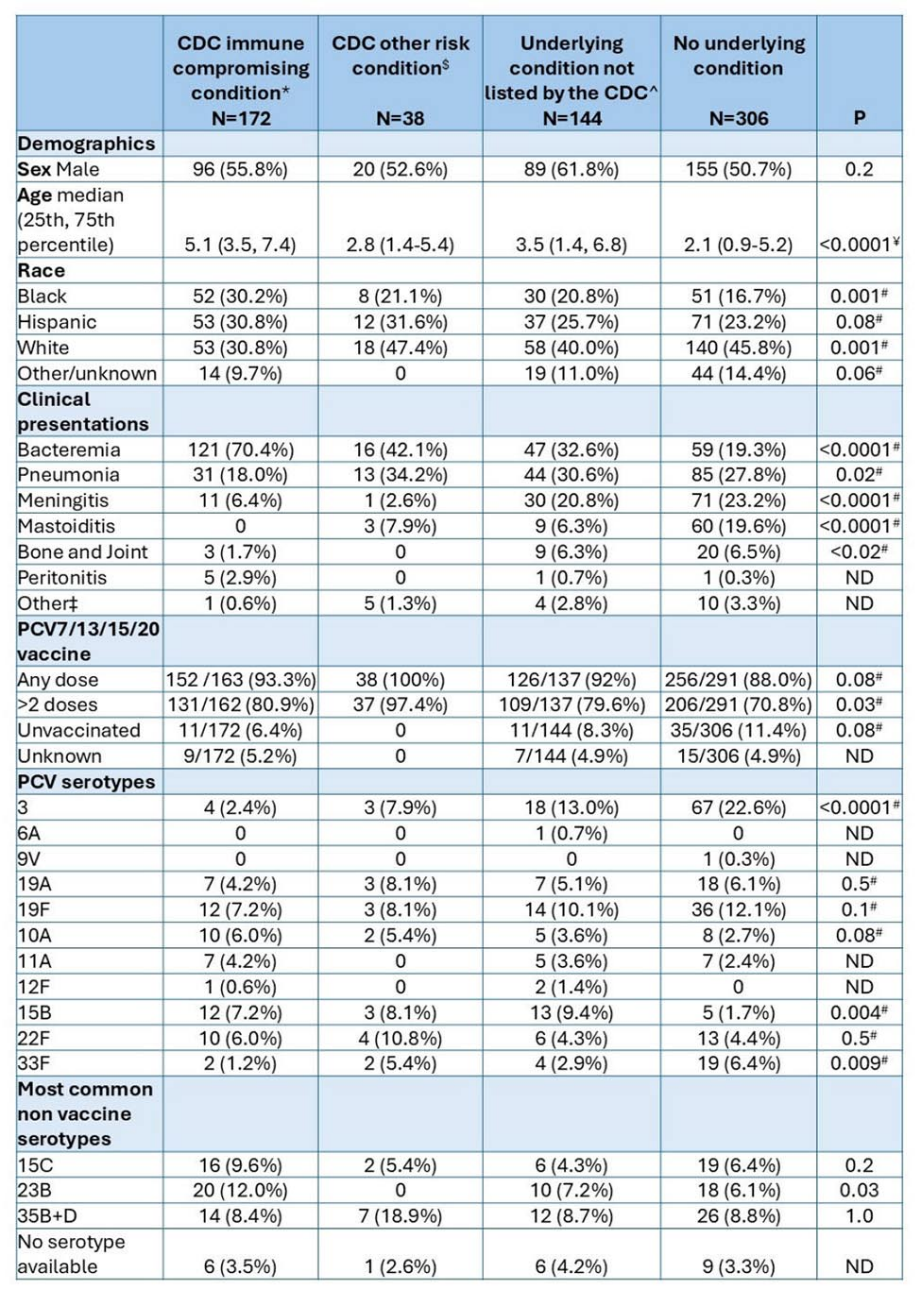
Demographic and clinical characteristics of the study population The CDC list of risk conditions: https://www.cdc.gov/pneumococcal/hcp/vaccine-recommendations/risk-indications.html#cdc_generic_section_5-risk-conditions: *e.g. nephrotic syndrome, asplenia, immunodeficiency, solid organ transplant; $e.g. cerebrospinal fluid leak, chronic heart, liver, kidney or lung disease; ^conditions not listed above e.g. mild asthma, certain genetic conditions; ‡Other disease presentations included orbital cellulitis or abscess (n=8), endocarditis (n=5), pericarditis (n=2), and one each of brain abscess, epidural abscess, endophthalmitis, and ascending cholangitis; #Fisher’s exact comparison of the CDC immunocompromising conditions risk group vs. the group of children without known underlying conditions; ¥ Wilcoxon rank sum comparison of the CDC immunocompromising conditions risk group vs. the group of children without known underlying conditions

**Methods:**

Data from an ongoing U.S. pediatric multicenter pneumococcal surveillance study were analyzed from 2017-2023. We serotyped the isolates and collected patient characteristics including IPD presentation, age, underlying conditions and PCV history. Statistical analysis was performed using STATA version16.

**Results:**

Among 660 cases with IPD, 210 had risk factors defined by the US Centers for Disease Control and Prevention (CDC) for the purpose of tailored vaccine directives and another 144 patients had other underlying conditions. (Table) Serotypes were available for 638 isolates. Differences among groups were observed for age, vaccine uptake, disease presentation and serotype distribution. Bacteremia was most common in patients with an immunocompromising condition and the most common serotype overall in this population was 23B (not contained in any licensed pediatric PCV). Serotypes 22F and 33F (both in PCV15 and PCV20) together caused 7% of cases; an additional 18% were of serotypes 10A, 11A, 12F and 15B (in PCV20). None of the unvaccinated patients in this group had a serotype 3 infection. Patients without underlying conditions were younger than the immune compromised, 50% presented with pneumonia or meningitis, and the most common serotype was 3. A higher percentage of children without CDC immunocompromising conditions presented with meningitis and mastoiditis.

**Conclusion:**

Our data show differences in clinical presentations and serotype distribution of IPD among children with or without known underlying conditions. PCV20 may provide additional protection for all groups and for the immunocompromised group, in particular, as 18% of their IPD isolates were serotypes not included in other licensed PCVs. Non-vaccine serotypes 15C, 23B, and 35B now are common causes of IPD in US children.

**Disclosures:**

Kristina G. Hulten, PhD, Pfizer: Grant/Research Support William J. Barson, MD, Pfizer: Grant/Research Support Jennifer Dien Bard, PhD, D(ABMM), FIDSA, bioMerieux: Advisor/Consultant|bioMerieux: Grant/Research Support Lindsay Grant, PhD, MPH, Pfizer: Employee|Pfizer: Stocks/Bonds (Private Company) Adriano Arguedas, Medical director, Pfizer employee: employee|Pfizer employee: Stocks/Bonds (Public Company) Maria J. Tort, PhD, Pfizer, Inc: Stocks/Bonds (Public Company) Ashley Miller, MA, CCRP, Pfizer: Stocks/Bonds (Public Company) Alejandro D. Cane, MD, PhD, Pfizer Inc.: All authors are employees of Pfizer Inc. and may hold stock and/or stock options of Pfizer Inc. Sheldon L. Kaplan, MD, Pfizer: Grant/Research Support

